# Post-COVID‑19-Arthritis. Manifestation unter dem klinischen Bild einer reaktiven Arthritis

**DOI:** 10.1007/s00393-021-01045-9

**Published:** 2021-07-09

**Authors:** H. Zeidler

**Affiliations:** Medizinische Hochschule Hannover, Carl-Neuberg Str. 1, 30625 Hannover, Deutschland

**Keywords:** Monarthritis, Oligoarthritis, HLA-B27, Nichtsteroidale Antirheumatika, Kortikosteroide, Monoarthritis, Oligoarthritis, HLA-B27, Nonsteroidal anti-inflammatory drugs, Corticosteroids

## Abstract

Es werden 13 Fallberichte einer reaktiven Arthritis im Zusammenhang mit einer Coronavirus-Krankheit-2019 (COVID‑19) referiert. Männer sind häufiger betroffen als Frauen. Die Arthritis manifestiert sich 4 bis 44 Tage nach der Infektion bzw. dem Auftreten der COVID‑19-Symptome. Die akute Arthritis ist monoartikulär oder oligoartikulär. Nur einer von 7 untersuchten Patienten war Humanes-Leukozyten-Antigen(HLA)-B27-positiv. Eine direkte virale Infektion des Gelenkes mit „severe acute respiratory syndrome coronavirus 2“ (SARS-CoV‑2) wurde in der Synovialflüssigkeit nicht nachgewiesen und in der Synovialis nicht untersucht. Die Arthritis wurde mit nichtsteroidalen Antirheumatika und/oder intraartikulären oder systemischen Kortikosteroiden erfolgreich behandelt. Die Pathogenese der post-COVID‑19-reaktiven Arthritis ist ungeklärt.

## Abstract (en)

A total of 13 case reports of reactive arthritis reported in the literature in connection with coronavirus disease 2019 (COVID‑19) are reviewed. Men were affected more frequently than women. The arthritis was manifested 4–44 days after the infection or the occurrence of the COVID‑19 symptoms. Acute arthritis was monoarticular or oligoarticular. Only 1 out of 7 patients examined was human-leucocyte-antigen(HLA)-B27 positive. A direct viral infection with severe acute respiratory syndrome coronavirus 2 (SARS-CoV-2) could not be detected in the synovial fluid and was not investigated in the synovial membrane. The arthritis was successfully treated with nonsteroidal anti-inflammatory drugs and/or intra-articular or systemic corticosteroids. The pathogenesis of post-COVID‑19 reactive arthritis is unknown.

Die aktuelle COVID‑19-Pandemie ist am häufigsten gekennzeichnet durch Manifestationen einer akuten Atemwegserkrankung mit interstitieller Lungenentzündung, Fieber, plötzlichem Verlust des Geruchs- und/oder Geschmackssinns, Müdigkeit und Kopfschmerzen. Muskuloskeletale Symptome in Form von Arthromyalgien werden in bis zu 50,4 % beobachtet [1]. Weitere Manifestationen sind Vaskulitis und das Vorhandensein von Autoantikörpern, die sonst bei Patienten mit rheumatischen Erkrankungen beobachtet werden. So führen rheumatische Symptome möglicherweise bei Patienten mit SARS-CoV-2-Infektionen („severe acute respiratory syndrome coronavirus 2“) zu einer Überweisung zum Rheumatologen und können dann eine entscheidende Rolle spielen bei der Identifizierung von SARS-CoV-2-Erkrankten zu Beginn oder im Verlauf. Im Folgenden wird, basierend auf einer Literatursuche in PubMed (Suchwörter: Covid‑19, arthritis, reactive arthritis), eine Übersicht gegeben über die im Zusammenhang mit einer COVID‑19-Infektion mitgeteilten Fallberichte einer reaktiven Arthritis (ReA).

## Publizierte Kasuistiken

Seit dem Auftreten der COVID‑19-Pandemie wurden bis April 2021 13 Fälle einer als ReA klassifizierten akuten Arthritis nach dieser Virusinfektion publiziert (Tab. 1; [2–14]). Männer sind häufiger betroffen als Frauen (*n* = 4/13). Neun der 13 Patienten waren älter als 45 Jahre. Die Arthritis manifestiert sich 4 bis 44 Tage nach der Infektion bzw. dem Auftreten der COVID‑19-Symptome.

**Table 1:** 

Studie	Alter (Jahre)/Geschlecht	Zeit vom Auftreten des COVID‑19-Symptoms bis zur Arthritis (Tage)	Befallene Gelenke	HLA-B27/Synoviaanalyse/RT-PCR der Synovialflüssigkeit für SARS-COViD‑19	Therapie der Arthritis	Verlauf
Ono et al. [2]	50/Mann	21	Beide Sprunggelenke, Enthesitis rechte Achillessehne	Negativ/leicht entzündlich/NU	NSAID und Kortikosteroid intraartikulär	Mäßige Besserung, keine weitere Beobachtung
Danssaert et al. [3]	37/Frau	12	Arthritis rechtes Handgelenk, im MRT Tendinitis mehrerer Strecksehnen	NU/NU/NU	Voltaren Gel® (Diclofenac; Novartis, Basel, Schweiz), Gabapentin, Hydromorphon	4 Wochen Nachverfolgung, Schmerzen und Druckschmerz gebessert, aber weiter Druckschmerz dorsal Hand und Handgelenk
Yokogawa et al. [4]	57/Mann	15	Linkes Handgelenk, rechtes Schultergelenk, beide Knie	NU/keine Kristalle/negativ	Keine	Remission spontan nach 27 Tagen
Liew et al. [5]	47/Mann	4 Tage nach Kontakt mit einem COVID‑19-Fall	Rechtes Knie, Balanitis mit geringem Erythem und Schwellung seiner Eichel	NU/keine Kristalle/PCR und Kultur auf COVID‑19 negativ	Etoricoxib, Triamcinolon intraartikulär	Keine Angabe
De Stefano et al. [6]	30/Mann	14	Rechtes Ellenbogengelenk, psoriatische Effloreszenzen Ellenbogen und Leisten	Negativ/NU/NU	NSAID	Remission nach 6 Wochen
Schenker et al. [7]	65/Frau	10 Tage nach Abklingen aller COVID‑19-assoziierten Symptome	Symmetrische Arthritis Handgelenke, Knie, Sprunggelenke, tastbare Purpura beider Waden	Positiv/NU/NU	Prednisolon	Schlagartige Rückbildung nach Beginn der Therapie mit Prednisolon
Mukarram et al. [8]	34/Mann	16	Rechtes Knie	NU/NU/NU	NSAID, Kortikosteroide intraartikulär	Komplette Rückbildung nach 10 Tagen
Hønge et al. [9]	53/Mann	18	Rechtes Knie, beide Sprunggelenke, linker Fuß	Negativ/viele Granulozyten, wenige Lymphozyten und keine Kristalle/NU	NSAID, Prednisolon	Nach 5 Tagen wieder gehfähig und Entlassung aus der stationären Behandlung
Di Carlo et al. [10]	55/Mann	44	Rechtes Sprunggelenk	Negativ/NU/NU	Methylprednisolon	Beschwerdefrei
Gasparotto [11]	60/Mann	32	Rechtes Knie und Sprunggelenk	Negativ/Leukozyten 20.000/mm^3^, Granulozyten 90 %, Monozyten 10 %/negativ	NSAID	Therapiedauer 4 Wochen
Parisi et al. [12]	58/Frau	25	Sprunggelenk, Tendinitis der Achillessehne	Negativ/Nu/NU	NSAID	Keine Schmerzen, aber nach 30 Tagen noch Synovitis im Ultraschall
Saricaoglu et al. [13]	73/Mann	22	Linke Zehe Grund- und Endgelenk, rechte 2. Zehe Interphalangeal- und Endgelenk	NU/NU/NU	NSAID	Komplette Remission
Jali [14]	39/Frau	3 Wochen	Fingermittelgelenk II plus III rechts, II links; Fingerendgelenk V beidseits	NU/NU/NU	Celecoxib	Komplette Remission nach 2 Wochen

*NSAID* nichtsteroidales Antiphlogistikum; *NU* nicht untersucht, *SARS-COVID-19* „acute respiratory syndrome coronavirus 2“

Die Gelenkbeteiligung ist monoartikulär oder oligoartikulär typisch für die ReA und mit überwiegendem Befall der unteren Extremität. Eine Enthesitis der Achillessehne wurde in 2 Fällen beobachtet [2, 12]. Bei einem Patienten mit Monoarthritis kam es gleichzeitig zu psoriatischen Effloreszenzen [6], und bei einem Humanes-Leukozyten-Antigen(HLA)-B27-positiven Patienten mit sehr hohen Anti-SARS-CoV-2-IgG-Antikörpertiter wurde als Hautbeteiligung eine tastbare Purpura beider Waden beobachtet [7]. Das HLA-B27 wurde bei 6 Patienten nicht bestimmt und war bei 1 von 7 untersuchten Patienten positiv [7]. Die Untersuchung der Synovialflüssigkeit mit reverser Transkriptase(RT)-PCR auf SARS-CoV‑2 erfolgte bei 2 Patienten und war beide Male negativ [5, 11]. Auch die bei einem Patienten durchgeführte Viruskultur auf SARS-CoV‑2 des Gelenkpunktates war negativ [5].

Die Arthritis ist bei einem Patienten am 27. Tag ohne Therapie abgeklungen [4]. Ein Patient war durch die Behandlung nur mäßig gebessert, wobei eine weitere Information zur Nachbeobachtung fehlt [2]. Bei einer Patientin bestand bei einer 4‑wöchigen Nachverfolgung weiter Druckschmerz der Hand und des Handgelenkes, und bei einer weiteren Patientin zeigte sich nach Abklingen der Schmerzen nach 30 Tagen noch eine Synovialitis im Ultraschall des Sprunggelenkes [12]. Bei den übrigen Patienten wurde die Arthritis mit nichtsteroidalen Antirheumatika und/oder intraartikulären oder systemischen Kortikosteroiden erfolgreich behandelt.

## Diskussion

Virale Arthritiden manifestieren sich üblicherweise mit einer symmetrischen Polyarthritis kleiner Gelenke, nur sehr selten als Monooligoarthritis und heilen meist spontan ab [15]. So wurden auch nach SARS-CoV-2-Infektionen vereinzelt akute Polyarthritiden beobachtet, v. a. mit positivem Antikörper gegen citrulliniertes Protein (ACPA), aber wohl eher zufällig und nicht in kausalem Zusammenhang [16–20]. Die akute, nach 4 Wochen remittierte Rheumafaktor-positive, ACPA-negative Polyarthritis einer 27-jährigen Frau dürfte eher in die zuvor erwähnten Post-COVID‑19-Arthritiden einzuordnen sein, auch wenn sie als ReA klassifiziert wurde [21].

Die 13 Fallbeschreibungen einer akuten, als ReA klassifizierten Arthritis weisen überwiegend die typischen klinischen Merkmale einer ReA auf. Nur die von Saricaoglu et al. [13] und von Jali [14] berichteten Fälle weichen vom Befallsmuster großer Gelenke ab mit einer Manifestation von Zehengelenken bzw. Fingergelenken. Patienten mit ReA können jedoch eine Arthritis in den oberen Gliedmaßen haben und eine leichte polyartikuläre Form, insbesondere in den kleinen Gelenken [22]. Auch das vollständige und schnelle therapeutische Ansprechen auf NSAID und/oder Kortikosteroide mit rascher Remission in den meisten Fällen sowie der für eine ReA typische zeitliche Zusammenhang sprechen für die mögliche ursächliche Beziehung mit der SARS-CoV-2-Infektion. Allerdings bleibt der kausale Zusammenhang ungeklärt, da in keinem Fall die Viren kulturell oder durch eine molekularbiologische Untersuchung im Gelenk identifiziert werden konnten. Nicht bei allen Patienten wurde umfassend mit mikrobiologischen (Blut‑, Urin- und Stuhlkulturen) und serologischen Tests das Spektrum der bakteriellen reaktiven Arthritis differenzialdiagnostisch abgeklärt. So ist neben der angenommenen viralen Genese nicht immer mit Sicherheit ausgeschlossen worden, dass sich zufällig eine ReA anderer Ursache manifestierte oder durch die SARS-CoV-2-Infektion ausgelöst wurde.

Für die Differenzialdiagnose ist wichtig, dass bei einer akuten Arthritis im Zusammenhang mit einer COVID‑19-Erkrankung ein Gelenkpunktat polarisationsoptisch auf Kristalle untersucht wird, da akute Krankheiten, einschließlich Infektionen, bekannte Risikofaktoren für Gicht- und Pseudogichtanfälle sind [23] und solche Fälle auch im Rahmen der COVID‑19-Pandemie beschrieben wurden [24].

Aktuell sind nur Vermutungen möglich zur Pathogenese der sich unter dem Bild einer ReA manifestierenden akuten Arthritis nach SARS-CoV-2-Infektionen. Eine direkte virale Infektion des Gelenkes wurde bisher in der Synovialflüssigkeit nicht nachgewiesen und in der Synovialis nicht untersucht. Diskutiert wird die Möglichkeit einer durch Immunkomplexe verursachten Entzündung, ohne dass jedoch bisher entsprechende Untersuchungen von Gelenkmaterial durchgeführt wurden [25]. In die Richtung einer möglicherweise immunkomplexvermittelten Genese weist der von Schenker et al. berichtete Fall, der über eine HLA-B27-positive Patientin mit kutaner Vaskulitis und sehr hohem Titer von Anti-SARS-CoV-2-IgG-Antikörpern als Zeichen einer sehr starken Immunantwort gegen das Virus berichtete [7].

Auch wenn wichtige Fragen des kausalen Zusammenhanges und der Pathogenese der Post-COVID‑19-ReA ungeklärt sind, dürfte die Kenntnis der Beobachtung solcher Fälle für den Rheumatologen wichtig sein, solange die COVID‑19-Pandemie nicht beendet ist.

## Fazit

- Reaktive Arthritis kann nach einer COVID‑19-Erkrankung auftreten.
- Die klinische und labortechnische Manifestation der nach SARS-CoV-2-Infektionen beobachteten reaktiven Arthritis ähnelt der durch andere Krankheitserreger verursachten reaktiven Arthritis.
- Differenzialdiagnostisch muss eine Gicht oder Chondrokalzinose ausgeschlossen werden.
- Nichtsteroidale entzündungshemmende Medikamente und Kortikosteroide wurden erfolgreich zur Behandlung eingesetzt.
